# Obstetrics and Gynæcology

**Published:** 1877-01

**Authors:** 


### IV. OBSTETRICS AND GYNÆCOLOGY.

1. *On the Weight of New Born Infants.* INGERSLEV. (*Revue des Sciences Médicales*, Oct. 10.)

The average weight of 3,450 infants at term, ascertained by the authorities of the lying-in asylum, at Copenhagen, was 3,333.5 grammes. Of this number 1,833 boys had an average weight of 3,380.6 grammes; and 1,617 girls showed an average weight of 3,279.7 gr. The boys therefore exceeded the girls in weight by an average of 100 gr. Of 19 children—13 boys and 6 girls—the weight varied between 9 and 10 pounds. One boy alone exceeded 10 pounds, weighing 10⅜.

1,723 children of primiparæ weighed, upon an average, 3,254 gr, and 1,727 children of multiparæ, 3,412 gr.; that is, the average weight of the latter was greater by 158 gr.

Mothers less than 19 years old seemed to bear lighter children than the others. Consulting one table in which the age of the mother is stated irrespective of the number of pregnancies, it is apparent that the weight of the newly born increases with that of the mother up to the 40th year. From the 40th to the 48th year the weight of the children again diminishes.

In order to ascertain the influence of a number of pregnancies, the author has prepared a table of the average weight of newly born children for the different pregnancies, irrespective of the age of the mother, indicating, however, the average epoch at which their pregnancies occurred. It is here apparent that the weight of the children increases with the number of pregnancies. There is, however, an exception in the case of girls born at the third pregnancy; but at the fourth and fifth their weight resumes its normal progression. Yet, since the age of the mother increases with the number of pregnancies, the increase in the weight of the child might be attributed to that circumstance.

After birth for a few days the infant loses its weight. Yet an increase may be noticed on the first day if the meconium is retained (which is ordinarily evacuated before the first weighing) or if the infant has been put to the breast. But this is a mere transitory increment, and the loss of weight is established at the second or third weighing. It is equivalent to one-fourteenth or one-fifteenth of the total weight, and is relatively and absolutely more considerable in the children of primiparæ, and in boys rather than girls. The latter, however, make up the loss more rapidly than the former. The initial loss is greater according as the child lacks vigor, and correspondingly the subsequent increase is more slow. The same is true in all children prematurely born.

The increment begins on the fourth (some authors say on the third) day. The causes of the loss of weight are: first of all, the evacuations; next, the condition of alimentation and assimilation. The meconium and the urine represent, at the most, the half of this loss; while the other must be produced by the operation of some other cause. It cannot be due to insufficient alimentation, since the richest alimentation cannot prevent it.

Note by the Eds.: The second factor referred to above as a cause of the loss of weight, must be the evaporation from the cutaneous surface. The unborn fœtus is an aquatic animal whose life is sustained while the body is immersed in a fluid medium of a high temperature. The effect of a sudden removal to a different medium of a different temperature is the evaporation of a large quantity of watery vapor from the surface and the moist tissues adjacent to it, as well from the skin as the lungs. It must be remembered that the pound referred to above, is the French metrical pound, equivalent to 500 grammes, or about 1 pound, 1 oz. and 10 drachms avoirdupois.

2. *Dangers of the Pessary.* Notta. (*L'Union Médicale*, Nov. 14.)

Several cases were reported. In the first, a woman 58 years old, afflicted with uterine prolapse (the organ projecting from the vulva) was immediately relieved by the application of the pessary. Soon after she fell accidentally, and the weight of the body came full upon the pessary. She suffered severe pain, which was followed in a few days by incontinence of urine. The pessary had penetrated into the bladder, and was withdrawn from the latter by Liston's forceps. Considerable loss of substance ensued and a permanent vesico-vaginal fistula.

A second woman, 35 years of age, had some insignificant disorder six months after the birth of her ninth child, for which she consulted a charlatan. He applied a Gariel pessary, which produced violent vaginitis with a very profuse and fetid muco-purulent discharge. The pain and the abundance and horrible odor of the discharge finally became insupportable, and as she found no relief from the advice of the would-be surgeon, she applied to Notta. He discovered an abscess which perforated the abdominal parietes, and was followed by an intractable fistula.

Another woman had granular endo-cervicitis with some hysterical symptoms, which improved under the author's cauterization and hydrotherapy, when by the advice of a neighbor she consulted the same charlatan. He applied the Gariel pessary, as usual, and produced a peri-uterine phlegmon, peritoneal inflammation, with abscesses, which in six months destroyed her life. Notta was summoned near the conclusion of the case and the unfortunate woman narrated to him the history of her sufferings with her own lips. Notta proceeded at once to institute judicial proceedings against the malfactor.

